# Don’t Always Prefer My Chosen Objects: Low Level of Trait Autonomy and Autonomy Deprivation Decreases Mere Choice Effect

**DOI:** 10.3389/fpsyg.2016.00524

**Published:** 2016-04-19

**Authors:** Zhe Shang, Tuoxin Tao, Lei Wang

**Affiliations:** Department of Psychology and Beijing Key Laboratory of Behavior and Mental Health, Peking UniversityBeijing, China

**Keywords:** mere choice effect, object evaluation, autonomy, self-enhancement, cognitive bias

## Abstract

Choice effect is a robust phenomenon in which even “mere choice” that does not include actual choosing actions could result in more preference for the self-chosen objects over other-chosen objects. In the current research, we proposed that autonomy would impact the mere choice effect. We conducted two studies to examine the hypothesis. The results showed that the mere choice effect measured by Implicit Association Test (IAT) significantly decreased for participants with lower levels of trait autonomy (Study 1) and when participants were primed to experience autonomy deprivation (Study 2). The theoretical and practical implications are discussed.

## Introduction

People make choices according to their preferences, indicating the important role that preferences play in choices. In addition, choice also has an impact on post-choice preferences. After the choice has been made, people’s liking of the chosen objects tends to increase while that of the rejected objects tends to decrease, known as the post-decisional spreading of alternatives (Brehm, 1956; Hammock and Brehm, 1966). In other words, people would prefer one of two similar objects simply because they chose one rather than the other, which is also known as the choice effect (Huang et al., 2009). Since the initial work of Brehm (1956), this phenomenon has got widespread attention (Ariely and Norton, 2008). The choice-induced preference has been found to exist strongly in several forms, such as in real choice actions (Patall et al., 2008) and illusory choices (Huang et al., 2009).

Over the past decades, the cognitive dissonance theory and self-concept related theories have been widely used in explaining the mechanism underlying the choice-induced preferences. The main classical explanation is based on the cognitive dissonance theory (Festinger, 1957), which argues that people are motivated to maintain internal consistency between cognitive inputs and behavioral outputs to reduce the uncomfortable feeling of dissonance. An individual is likely to experience cognitive dissonance if he/she holds negative attitudes toward an object, given that the object has been chosen by oneself, because thus a conflict would occur between the cognitive input (“I don’t like this thing”) and the behavioral output (“I chose this thing”) (Van Overwalle and Jordens, 2002). In order to reduce this uncomfortable feeling of cognitive dissonance, individuals would increase their liking of the chosen objects when the actual choice action has been taken (Olson and Stone, 2005). This theory helps to explain why people prefer a chosen object to an unchosen one simply because they took an explicit action to choose the object. However, when there is no explicit choosing action, the cognitive dissonance theory loses its power in explaining the mechanism of the choice-induced preference. Recent empirical research has indicated that the choice effect happens even when individuals lack the awareness of their choosing behaviors (Lieberman et al., 2001; Coppin et al., 2010), suggesting that cognitive dissonance may not be a necessary prerequisite of choice-induced preference. Indeed, choice-induced preferences are found even when the choices were seemingly trivial (Langer and Rodin, 1976) or wholly illusory (Langer, 1975). That is, choice-induced preferences existed even when there was no explicit choice action and thus the awareness of cognitive dissonance may not be present. The phenomenon where choice itself is powerful enough to induce liking, even in the condition that choosing is illusory and does not actually occur, was termed by Huang et al. (2009) as the mere choice effect.

The theory referring to the positive valence of self node helps to explain the choice effect as well (Greenwald et al., 2002). “Self node” means that self was treated as a node in the self-related concept tree. “Self” is the sum of all that one can call his/her own (James, 1890). “My choice” is also a part of the self-concept. Theories and phenomena associated with self-serving or self-protecting biases (Sedikides and Strube, 1997), such as self-enhancement (Kurman, 2001), self-affirmation (Brown and Dutton, 1995), and self-verification (Chen et al., 2006) imply that people are prone to evaluate “my choice” as better than “others’ choice” to maintain a positive self-image, and thus would display a positive evaluation on self-chosen objects. “Self node” affects the choice-preference link by increasing preference on self-chosen objects in an implicitly way, which leads to mere choice effect. On the other hand, to choose is to express a preference and to assert the self (Leotti et al., 2010). Consequently, attaching a high evaluation to “my choice” implies acceptance of the self and thus in turn brings higher self-satisfaction and self-esteem. The scope of self-concept is broader than just one’s possessions or decisions (choices). As the Ryan and Deci (2000) theorized self-determination theory (SDT), the need for competence, autonomy, and psychological relatedness are three psychological needs that motivate the self to initiate behavior (Deci and Ryan, 1985, 2000). The act of self-regulation, such as autonomy, is also related to self-concept. Experiencing autonomy promotes the sense that an individual’s behavior is self-motivated and self-determined and thus maintain a positive self-image. Applied in the objects evaluation, another possible theoretical explanation refers to the role that autonomy plays in the choice effect.

The sense of autonomy refers to the extent to which people feel free to make their own decisions and experience a sense of volition in their actions (van Prooijen, 2009). Choosing behavior increases the experience of autonomy by allowing people to exert their right to make a decision. Previous research has demonstrated that people evaluate the chosen alternative as more desirable than the rejected alternative, in order to reassert their autonomy (Hammock and Brehm, 1966). Experiments have suggested that manipulations designed to enhance one’s experience of autonomy can boost intrinsic motivation and energize behavior (Swann and Pittman, 1977; Zuckerman et al., 1978; Simon and McCarthy, 1982, Unpublished). Offering people an optimal amount of choice enhanced their intrinsic motivation and energy to persist (e.g., deCharms, 1968; Deci and Ryan, 1985). As demonstrated by plenty of research, autonomy is associated with intrinsic motivation (Deci et al., 1999), persistence (Moller et al., 2006), goal attainment (Sheldon and Elliot, 1998), and creativity (Sheldon, 1995), indicating that autonomy elicits positive outcomes. Additionally, perceived autonomy has an effect on enhancing happiness (Chekola, 2007; Demir et al., 2011), job satisfaction, and a general increase in subjective well-being (Sheldon et al., 2004), all of which conclude that autonomy elicits positive personal feelings. Preference for an object represents the positive objective valence that one attaches to the object in the process of evaluation. As has been validated by previous research, personal positive state and feelings influence evaluation, in terms of increasing personal preference/sensitivity on surroundings and targeted objects (Gu et al., 2010; Yang et al., 2014). It is possible that the sense of autonomy elicited by choosing enhances an individual’s evaluation toward an object, because experiencing autonomy induces positive feelings, which in turn have a positive effect on the evaluation of the object.

We speculate that the experience of autonomy may enhance the preference on self-chosen objects. Thus, we measured the relationship of trait autonomy and choice effect in Study 1 and propose the hypothesis: (1) trait autonomy is positively correlated with choice effect. We infer that autonomy may moderate the choice-preference link. When an individual takes a choosing action or is simply acknowledged that something has been chosen by himself/herself, the sense of autonomy is generated, which brings him/her positive feelings. These positive feelings in turn may enhance his/her positive evaluation to the surroundings. On the contrary, the lack of autonomy may reduce the preference for self-chosen objects. To our best knowledge, however, no study has provided empirical evidence for the role of autonomy in the choice effect. In Study 2, we investigated the influence of different levels of autonomy experience on the choice effect by using a priming task to set three conditions: the autonomy fulfillment condition, the autonomy deprivation condition, and the control condition. Here we propose the hypothesis: (2) the choice effect would occur in the autonomy fulfillment condition and in the control condition, but not in the autonomy deprivation; (3) autonomy deprivation would decrease or even eliminate the choice effect when compared with the autonomy fulfillment condition; (4) autonomy deprivation would decrease or even eliminate the choice effect when compared with the control condition; (5) autonomy fulfillment would increase the choice effect when compared with the control condition.

### Overview of Two Studies

In order to study the influence of autonomy on the choice effect while excluding the impact of cognitive dissonance, we employed a modified illusory choice paradigm, adapted from Huang et al. (2009) to measure the presence of the mere choice effect. We adopted the Implicit Association Test (IAT, Greenwald et al., 1998) that records the response time when participants respond to settled categories of objects framed by positive or negative adjectives.

In measuring autonomy, we treated it as an individual difference variable (Deci and Ryan, 2000). It can be either dispositional or situational. Thus, we tested our hypothesis through two studies. In Study 1, we recorded participants’ self-report trait autonomy and divided participants into high and low autonomy groups accordingly. Study 2 adopted a priming paradigm to manipulate the situational autonomy in three levels.

## Study 1: The Relationship Between Trait Autonomy (Between-Subject Variable) and the Mere Choice Effect

In Study 1, we used a scale to measure trait autonomy as an individual differential variable. Subsequently, we tested the mere choice effect using an IAT paradigm. We then calculated the relationship of trait autonomy and the mere choice effect.

### Materials and Methods

#### Participants

A total of 91 graduate and undergraduate students (50 female, 41 male, average age = 22.2 years, *SD* = 2.39, ranging from 19 to 25 years old) participated in the experiment for a cash reward (US$2). We asked all the participants to conduct an object chosen task in which they would see some texts in a computer screen and react by push some buttons on the keyboard. Each of them wrote informed written consent before the test. They were told there would be no any dangers while they were doing the experiment. They were told their rights and they can decide to or not to participate in this experiment, and they had the right to quit the experiment at any time of the experiment. This study was in accordance with the Declaration of Helsinki and was approved by the Ethics Committee of the Department of Psychology, Peking University.

#### Procedure and Materials

Participants were told that there were two unrelated tasks. After each experiment session, we asked the participant whether he/she thought the two parts were related. None of them replied yes. In the first task, they were required to complete questionnaires including trait autonomy and demographic survey. The second task was a mere choice task presented on computer, adapted from Huang et al. (2009), which created a mere choice situation to the participants. As previous research has demonstrated (Huang et al., 2009), when participants are asked to choose something for a third party, they would implicitly prefer the self-chosen object to the other-chosen objects (i.e., the choice effect).

#### Trait Autonomy

The five-item Choicefullness Subscale of the Self Determination Scale (Sheldon, 1995; Sheldon et al., 1996) was used to measure trait autonomy. Each item presented participants with two opposing statements. Participants were asked to indicate which of the two statements was more appropriate for describing themselves. An example item is showed as follows: “I always feel like I choose the things I do” (Statement A) versus “I sometimes feel that it’s not really me choosing the things I do” (Statement B) (5-likert scale: 1 = *only A feels true*; 5 = *only B feels true*). The answers were coded such that lower scores indicated lower level of autonomy. Our data showed good internal consistency (Cronbach’s α = 0.697)

#### Mere Choice Effect

To evaluate the mere choice effect (an affect reflected the degree of preference on self-chosen objects over other-chosen objects), both of the two studies used a modified illusory choice paradigm developed by Huang et al. (2009), in which participants were asked to imagine a scenario about choice instead of taking an actual choice action.

This part was completed on computers using the Inquisit laboratory software.

In Step 1, participants read the following two-page scenario on the computer screen:

*Please visualize the following scenario. You and your friend (marked as* ***the other*** *in the experiment) bought six products in a supermarket for another friend: a mug, a small figurine, a piece of chocolate, a piece of candy, a pen, and a ruler. Please visualize and remember these products. They will be used in the following experiment (Page 1).*

Among these six products, three of them were chosen by you, and the other three were chosen by the friend (the other) shopping with you. You chose the mug, the chocolate, and the pen. Your friend chose the small figurine, the candy, and the ruler. Please spend 2 min to visualize and separately remember your choices and your friend’s choices. They will be used in the following experiment (Page 2).

Half of the participants were shown the aforementioned scenario. The other half read similar instructions except that we swapped the products assigned to the self and the other.

Then the participants began the modified illusory choice IAT (Huang et al., 2009). This IAT followed the procedure designed by Greenwald et al. (1998), involving two target categories (objects chosen by the self vs. objects chosen by the other) and two attribute categories (positive vs. negative). Target categories followed the scenario described previously. The attribute categories were previously used in many studies (Maison et al., 2004; Huang et al., 2009). The positive stimuli included the Sun, luck, love, fun, happiness, pleasure, holiday, and friendship. The negative stimuli included disease, death, murder, accident, poison, war, tragedy, and vomit. Our study was consistent with the classical IAT paradigm (Lane et al., 2007), target words and attribute words were presented together in the IAT paradigm. In the two main tasks of IAT, there were two situations: in one situation, the words “self-chosen objects/positive attributes” appeared in the top left-hand corner while the words “other-chosen objects/negative attributes” appeared in the top right-hand corner; in the other situation, the words “self-chosen objects/negative attributes” appeared in the top left-hand corner while the words “other-chosen objects/positive attributes” appeared in the top right-hand corner. As can be seen, in both situations, the self-chosen and other-chosen objects appeared together as the target words, making it impossible to analyze their effects separately.

The IAT consisted of five classification tasks (see **Table 1**): attribute discrimination task (Block 1, 24 trials), initial target-category discrimination task (Block 2, 24 trials), initial combined task (Block 3, 24 trials for practice, and Block 4, 48 trials for data collection), reversed target-category discrimination task (Block 5, 48 trials), reversed combined task (Block 6, 24 trials for practice and Block 7, 48 trials for data collection).

**Table 1:** Task process of the IAT paradigm in Studies 1 and 2.

			Response key assignment	
			
Block	Task	Trials	Left key	Right key
1	Attribution discrimination	24	Positive	Negative
2	Initial target discrimination	24	Objects chosen by the self	Objects chosen by the other
3	Initial combined task	24	Positive; objects chosen by the self	Negative; objects chosen by the other
4	Initial combined task	48	Positive; objects chosen by the self	Negative; objects chosen by the other
5	Reversed target discrimination	48	Objects chosen by the other	Objects chosen by the self
6	Reversed combined task	24	Positive; objects chosen by the other	Negative; objects chosen by the self
7	Reversed combined task	48	Positive; objects chosen by the other	Negative; objects chosen by the self


In the attribute discrimination task (Block 1, 24 trials), participants were asked to press a left key (F) when a positive word appeared on the screen and a right key (J) for a negative word. Similarly, in the initial target-category discrimination task (Block 2, 24 trials), objects chosen by the self (responding by pressing the left key) and objects chosen by the other (responding by pressing the right key) were discriminated. In the initial combined task (Block 3, 24 trials for practice and Block 4, 48 trials for data collection), attribute and target discrimination trials were combined and participants had to press the left key when either a positive word or an object chosen by the self was presented and the right key when a negative word or an object chosen by the other was presented (the compatible condition, we replicated the IAT paradigm in accordance with a previous study (Huang et al., 2009), in which the participants’ responses showed that self-chosen objects were implicitly linked with positive words (e.g., happiness, sunshine), as opposed to negative words (e.g., death, war), and in which other-chosen objects were implicitly linked to negative words, as opposed to positive words. Thus, we argue that the compatible condition was composed of self-chosen objects with positive descriptions and other-chosen objects with negative descriptions, just as Huang et al., 2009 showed). In the reversed target-category discrimination task (Block 5, 48 trials), Block 2 was repeated with a switch of the categorization keys by pressing left key when an object chosen by the other appeared on the screen and a right key when an object chosen by the self appeared. The reversed combined task (Block 6, 24 trials for practice and Block 7, 48 trials for data collection) again combined two individual tasks. Participants were instructed to press the left key when either a positive word or an object chosen by the other was presented and press the right key when a negative word or an object chosen by the self was presented (incompatible condition). Each block started with a brief instruction for the following task and a request to respond as fast as possible while trying to minimize mistakes. Participants were also reminded that their error rate and response times would be recorded.

Different random orders of trails were used for different participants. Half of the participants went through the seven blocks in the order presented previously; to remove any order effect, Blocks 2, 3, and 4 were swapped with Blocks 5, 6, and 7 for the other half of the participants. Only data from Blocks 4 and 7 were used for analysis. Each block started with a brief instruction.

After each experiment session, the participant was fully debriefed, thanked, and paid for his/her participation.

### Results

We analyzed the data following the processes suggested by Greenwald et al. (1998). The first two trials of each block were excluded since the response latencies for them were typically longer. Next, we recoded the latencies by excluding reaction times (RTs) that were below 300 ms or above 3000 ms, so that we could control for outlying trials where distraction and anticipation likely affected the trial. We disregarded any participant with an error rate above 30%. Thus, our final data analysis included 87 participants (46 female, 41 male, average age = 21.1 years, *SD* = 2.36, ranging from 18 to 25 years old).

In the IAT task, the compatible condition was composed of self-chosen objects with positive descriptions and other-chosen objects with negative descriptions, while the incompatible condition was composed of self-chosen objects with negative descriptions and other-chosen objects with positive descriptions. The choicer-attitude valence compatible level (the compatible condition and the incompatible condition) was a within-subject variable. We conducted a one-way repeated ANOVA of choice-attitude valence compatibility level (compatible condition vs. incompatible condition), after controlling for gender and age. Results showed a significant main effect, *F*(1,86) = 4.023, *p* < 0.05, η^2^ = 0.046. Participants’ RT in the compatible condition (*M*_RT_ = 753 ms, *SD* = 206 ms) was faster than that in the incompatible condition (*M*_RT_ = 882 ms, *SD* = 214 ms). We suggest that participants preferred the self-chosen objects with positive descriptions and other-chosen objects with negative descriptions over other-chosen objects with positive descriptions and self-chosen objects with negative descriptions. In other words, compared with perceived other-chosen objects, perceived self-chosen objects were more strongly associated with positive than with negative words, indicating that people implicitly preferred self-chosen objects to other-chosen objects, despite their lack of actual experience of a choosing process, namely the mere choice effect. This result is consistent with the previous study of Huang et al. (2009).

#### Choice Effect and Autonomy

We used the difference response time (d-RT). It is the RT in the incompatible condition (other-chosen objects that were implicitly linked with positive words and self-chosen objects that were implicitly linked with negative words) minus the RT in the compatible condition (self-chosen objects that were implicitly linked with positive words and other-chosen objects that were implicitly linked with negative words) as the indicator of the choice effect (*Mean different RT* = 129 ms, *SD* = 174 ms). Longer d-RT indicated a larger choice effect while shorter d-RT indicated a smaller choice effect. In the meantime, lower scorers on the five-item Choicefulness Subscale of the Self Determination Scale indicated lower level of trait autonomy, and higher scores indicated higher level of trait autonomy. The mean score of trait autonomy was 14.78, and standard deviation was 3.289.

Hypothesis 1 proposed that trait autonomy is positively correlated with choice effect. We examined the effect of trait autonomy on choice effect by controlling for gender and age in a hierarchical analysis. We conducted a hierarchical regression analysis by entering gender in a first block/model, age in a second block/model, and the trait autonomy as the independent variable in a third block/model. All variables were normalized as *Z*-scores for data analysis. The regression coefficients, standard error, 95% confidence interval [CI], the change in *F* statistic (including *p*-value), and the coefficient of determination change (delta *R*^2^) for each model are shown in **Table 2.** The results of regression analysis showed that after controlling for gender and age, the β of trait autonomy on choice effect represented by d-RT in the IAT task was 0.341, (*SE* = 0.105, *p* < 0.01, 95% confidence interval [CI] = [0.132, 0.551]), which suggested a significant direct effect. **Table 2** shows that trait autonomy explained incremental variance of d-RT in IAT (11.1%), *p* < 0.01, suggesting that people with a higher level of trait autonomy showed a larger choice effect. It should be noted that only the effect of trait autonomy on choice effect was obtained; the other two variables (gender, age) were not significant predictors of the criteria. This above-mentioned result provides support for Hypothesis 1.

**Table 2:** The hierarchical regression of predictors on choice effect in Study 1.

Predictor	The d-RT in IAT								
	
	95% CI (Confidence interval)	*R*	*R*^2^	Δ*R*^2^	*F*	*p*	β	*SE*	*p*
Model 1		0.105	0.011	0.011	0.945	0.334			
Gender	[-0.110, 0.319]						0.105	0.108	0.334
Model 2		0.111	0.012	0.001	0.121	0.729			
Gender	[-0.108, 0.325]						0.109	0.109	0.332
Age	[-0.254, 0.179]						-0.038	0.109	0.729
Model 3		0.351	0.123	0.111^∗∗^	10.501^∗∗^	0.002			
Gender	[-0.129, 0.284]						0.078	0.104	0.456
Age	[-0.313, 0.106]						-0.104	0.105	0.327
Trait autonomy	[0.132, 0.551]						0.341^∗∗^	0.105	0.002


**N* = 87. ^∗∗^*p* < 0.01.*

## Study 2: The Influence of Autonomy on the Choice Effect

Study 2 is designed to extend the results of Study 1. According to the findings in Study 1, there is a positive correlation between trait autonomy and the choice effect. To further investigate the nature of this relationship, we examined whether the choice effect would remain when autonomy was deprived in a between-subject design. We aimed to test whether experimentally manipulated autonomy affects the choice effect. We repeated the steps of Study 1, except that we did not measure trait autonomy by questionnaire but manipulated the level of autonomy. In this study, the perceived autonomy was manipulated by a priming task, which comprised: an autonomy fulfillment condition, an autonomy deprivation recall condition, and a control condition.

### Materials and Methods

#### Participants

Sixty-five participants (38 women, 27 men; *M*_age_ = 22.3, *SD* = 1.9, range from 18 to 27 years old), all of them were university students. They were randomly assigned to the three experimental conditions. Twenty-two participants were assigned in the autonomy-fulfillment condition, 21 participants were assigned in the autonomy-deprived condition, and 21 participants were assigned in the control condition. All the participants were informed of conducting an object chosen task during the recruitment and before the experiment. Informed written consent was obtained from each participant before the test. They were told there would be no any dangers while they were doing the experiment in which they would see some texts in a computer screen and react by push some buttons on the keyboard. They were told their rights and they can decide to or not to participate in this experiment, and they had the right to quit the experiment at any time of the experiment. They were rewarded for about 2 US dollars for their participation. This study was in accordance with the Declaration of Helsinki and was approved by the Ethics Committee of the Department of Psychology, Peking University.

#### Procedure and Materials

Participants were told that the session consisted of two separate tasks. The first task was introduced as focusing on the recall of past events, while the real purpose of it was to prime the sense of autonomy in participants. For example, participants in the autonomy fulfillment condition were asked to write an essay about a particular incident in which they felt high level of autonomy. The introduction went as follows:

#### Manipulating Materials of Autonomy Experience

##### Autonomy-fulfillment condition

“*The first part of this session is story collection. Please describe an event about personal autonomy. Here, the autonomy is defined as when an individual is able to make their own choices freely, and experiences a sense of control over their decisions. (If you have any questions about this definition, please ask the experimenter.)*

*Now, please write down an event based on your real experience, in which your autonomy was satisfied. Please elaborate the details as much as possible, including the objective circumstances and your subjective feelings.*”

##### Autonomy-deprivation condition

“*The first part of this session is story collection. Please describe an event about personal autonomy. Here, the autonomy is defined as when an individual is able to make their own choices freely, and experiences a sense of control over their decisions. (If any questions about this definition, please ask the experimenter.)*

*Now, please write down an event based on your real experience, in which your autonomy was deprived. That is to say, your behaviors were not completely controlled by yourself and some decisions were not self-decided. Please elaborate the details as much as possible, including the objective circumstances and your subjective feelings.*”

After completing the recall task, participants completed the Choicefulness subscale of the Self Determination Scale (Sheldon, 1995; Sheldon et al., 1996), as a manipulation check of the autonomy priming. For the participants in the control condition, they were given no priming materials and completed the scale directly. Then, all the participants were introduced to what ostensibly was a second task: the IAT task, which was the same task as in Study 1. At last, participants were fully debriefed, thanked, and paid for their participation.

### Results

After applying the same data protocol used in Study 1, the final data analysis of Study 2 included 64 participants (37 women, 27 men; *M*_age_ = 21.7, *SD* = 1.95, range from 18 to 26 years old).

#### Manipulation Checks

For the manipulation check of autonomy priming, a one-way ANOVA revealed that there was a significant main effect of the autonomy manipulation (the autonomy fulfillment condition, the autonomy deprivation condition, the control condition) on the Choicefulness Scale scores, *F*(2,61) = 3.190, *p* = 0.048, η^2^ = 0.044. After controlling for gender and age, the ANOVA showed that there was no significant difference of Choicefulness Scale scores between the autonomy fulfillment condition (*M* = 16.86, *SD* = 2.624) and the control condition (*M* = 15.67, *SD* = 2.850), *F*(1,42) = 2.012, *p* = 0.164, η^2^ = 0.048. While participants scored significantly higher (*M* = 15.67, *SD* = 2.850) on the Choicefulness Scale in the control condition than in the autonomy deprivation condition (*M* = 14.71, *SD* = 2.918), *F*(1,41) = 1.109, *p* = 0.299, η^2^ = 0.028. Participants scored significantly higher (*M* = 16.86, *SD* = 2.624) in the autonomy fulfillment condition than those in the autonomy deprivation condition (*M* = 14.71, *SD* = 2.918), *F*(1,42) = 6.194, *p* = 0.017, η^2^ = 0.137. This result confirmed the validity of autonomy manipulation in autonomy fulfillment condition and autonomy deprivation condition. That is, compared with those in autonomy deprivation condition, participants in autonomy fulfillment condition experienced a higher level of autonomy (see **Table 3**).

**Table 3:** Autonomy scores in autonomy priming task’s manipulation check in Study 2.

	*N*	Mean scores	*SD*
Autonomy fulfillment	22	16.86	2.62
Control condition	21	15.67	2.85
Autonomy deprivation	21	14.71	2.92


#### Autonomy and the Choice Effect

**FIGURE 1:**
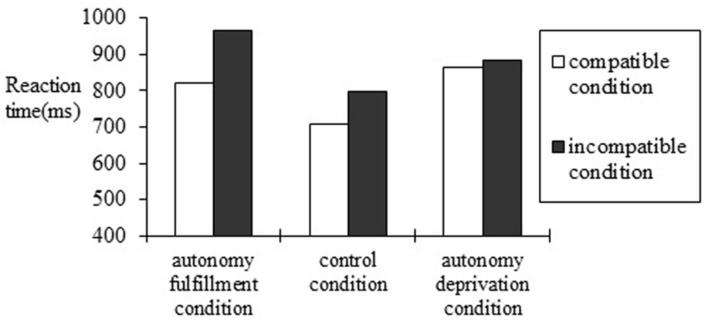
**Reaction time of compatible and incompatible evaluation conditions under the autonomy fulfillment, autonomy deprivation, and control condition in Study 2**.

To further identify how autonomy affects RT in compatible condition and incompatible condition, we analyzed a 3 (autonomy priming manipulation: the autonomy fulfillment condition, the control condition, and the autonomy deprivation condition) by 2 (choice-attitude valence compatibility level: the compatible condition and the incompatible condition) mixed design, in which the autonomy priming manipulation was a between-subject variable and the choice-attitude valence compatible level was a within-subject variable. A two-way repeated ANOVA of autonomy priming manipulation and choicer-attitude valence compatible on response time was conducted, controlling for gender and age (see **Figure 1**). The results showed a main effect: autonomy priming manipulation was significant, *F*(2,61) = 5.341, *p* = 0.007, η^2^ = 0.15. The response time in the autonomy fulfillment group (*M*_RT_ = 892 ms, *SD* = 45 ms) was significantly longer than that in the control group (*M*_RT_ = 713 ms, *SD* = 46 ms), *F*(1,42) = 7.406, *p* = 0.01, η^2^ = 0.155. The response time in the autonomy deprivation group (*M*_RT_ = 906 ms, *SD* = 46 ms) was also significantly longer than that in the control group (*M_RT_* = 713 ms, *SD* = 46 ms), *F*(1,41) = 8.477, *p* = 0.006, η^2^ = 0.174. The response time in the autonomy deprivation group (*M*_RT_ = 906 ms, *SD* = 46 ms) was not significantly longer than that in the autonomy fulfillment group (*M*_RT_ = 892 ms, *SD* = 46 ms), *F*(1,42) = 0.142, *p* = 0.709, η^2^ = 0.003. These results indicated that autonomy priming (whether fulfillment or deprivation) led to slower participant RTs. A main effect of choice-attitude valence compatibility level on response time was significant, *F*(1,61) = 11.877, *p* = 0.001, η^2^ = 0.15. The response time of incompatible trials (*M* = 882 ms, *SD* = 28 ms) was significantly longer than that of compatible trials (*M* = 793 ms, *SD* = 28 ms), indicating the conflict of objects and adjectives in the incompatible condition.

The interaction of priming manipulation and compatibility level was significant, *F*(2,61) = 4.550, *p* = 0.015, η^2^ = 0.11, indicating that the response time was modulated by the priming manipulation. *Post hoc* analyses showed that, in the autonomy fulfillment condition, participants responded significantly faster in compatible condition (*M* = 819 ms, *SD* = 237 ms) than in the incompatible condition (*M* = 964 ms, *SD* = 216 ms), *F*(1,21) = 6.004, *p* = 0.024, η^2^ = 0.219, indicating the existence of the mere choice effect. For participants in the control condition, they responded significantly faster in the compatible condition (*M* = 663 ms, *SD* = 189 ms) than in the incompatible condition (*M* = 766 ms, *SD* = 251 ms), *F*(1,20) = 8.787, *p* = 0.008, η^2^ = 0.255, which also indicates the presence of the mere choice effect. These two findings demonstrated that participants preferred the self-chosen objects with positive descriptions (e.g., happiness, sunshine) and other-chosen objects with negative descriptions (e.g., death, war) over other-chosen objects with positive descriptions and self-chosen objects with negative descriptions. People implicitly preferred self-chosen objects to other-chosen objects even without owning those objects. The mere choice effect occurs, even without actually experiencing a choosing process.

For participants in the autonomy deprivation condition, their RT in the compatible condition (*M* = 897 ms, *SD* = 259 ms) was not significantly different from that in the incompatible condition (*M* = 915 ms, *SD* = 198 ms), *F*(1,20) = 2.111, *p* = 0.163, η^2^ = 0.086, suggesting no mere choice effect. Here, the effect did not occur because there was no significant difference between compatible condition and the incompatible condition. The reference point is the participants’ RT in the compatible condition, and we compare this reference RT with RT in the incompatible condition. The choice effect appeared in the autonomy fulfillment condition and the control condition, but not in the autonomy deprivation condition. The result is consistent with our Hypothesis 2.

We also used the difference response time (d-RT) as the indicator of the choice effect. The longer d-RT represents the larger choice effect. We conducted a one-way ANOVA of priming perceived autonomy on choice effect indicated by d-RT in the IAT task, controlling for gender and age. As hypothesized, the main effect of perceived autonomy was significant, *F*(2,61) = 4.550, *p* = 0.015, η^2^ = 0.11.

We conducted the planned contrasts. By setting contrast coefficients, we can not only compare two means at once, but also combine multiple means from different levels to compute mean pair tests in these contrasts. Planned contrasts revealed that priming autonomy fulfillment (*M*_d-RT_ = 145 ms, *SD* = 165 ms) significantly increased the choice effect compared to priming autonomy deprivation (*M*_d-RT_ = 18 ms, *SD* = 170 ms), *t*(61) = 2.698, *p* = 0.009, *d* = 0.843, indicating a significantly larger choice effect in the autonomy fulfillment condition than in the autonomy deprivation condition. Compared with autonomy fulfillment, autonomy deprivation decreased the choice effect. This result is consistent with Hypothesis 3. Participants primed in the control group (*M*_d-RT_ = 103 ms, *SD* = 123 ms) did not have a significantly larger choice effect compared to participants primed with autonomy deprivation (*M*_d-RT_ = 18 ms, *SD* = 170 ms), *t*(61) = 1.785, *p* = 0.075, *d* = 0.564, the trend did not reach significance. Here, we did not find supporting evidence for Hypothesis 4, which proposed that autonomy deprivation would decrease the choice effect when compared with the control condition. Participants primed with autonomy fulfillment (*M*_d-RT_ = 145 ms, *SD* = 165 ms) did not show a significantly larger choice effect compared to participants in the control group (*M*_d-RT_ = 103 ms, *SD* = 123 ms), *t*(61) = 0.892, *p* = 0.376, *d* = 0.279; the trend did not reach significance. Among the above effect sizes, the first one (i.e., the choice effect in the perceived autonomy fulfillment group compared to the autonomy deprivation group) is a fairly large effect. For Hypothesis 5, which stated that autonomy fulfillment would increase the choice effect when compared with the control condition, we neither found statistical support (see **Table 4**).

**Table 4:** The d-RT in IAT in autonomy priming conditions in Study 2.

	*N*	Mean d-RT (ms)	*SD* (ms)
Autonomy fulfillment	22	145	165
Control condition	21	103	123
Autonomy deprivation	21	18	170


Similar to the data analysis procedure in Study 1, we examined the effect of autonomy priming on choice effect (indicated by the d-RT) in a regression model after controlling for gender and age. We entered gender in a first block/model, age in a second block/model, and the autonomy priming (score 3 represented autonomy fulfillment, score 2 represented control group, score 1 represented autonomy deprivation) as the independent variable in a third block/model. All variables were normalized as *Z*-scores for data analysis. The regression coefficients, standard error, 95% confidence interval [CI], the change in *F* statistic (including *p* value), and the coefficient of determination change (delta *R*^2^) for each model are shown in **Table 5.** The results of regression analysis showed that after controlling for gender and age, the β of autonomy priming on choice effect represented by d-RT in the IAT task was.330, (*SE* = 0.116, *p* < 0.01, 95% confidence interval [CI] = [0.097, 0.563]), which suggested a significant direct effect. **Table 5** shows that autonomy priming explained incremental variance of d-RT in IAT (10.8%), *p* = 0.006, suggesting that participants with autonomy fulfillment showed a larger choice effect and supporting Hypothesis 2 (see **Table 5**).

**Table 5:** The hierarchical regression of predictors on choice effect in Study 2.

Predictor	The d-RT in IAT							
	
	95% CI (Confidence interval)	*R*	*R*^2^	Δ*R*^2^	*F*	*p*	β	*SE*	*p*
Model 1		0.136	0.019	0.019	1.174	0.283			
Gender	[-0.388 0.115]						-0.136	0.126	0.283
Model 2		0.285	0.081	0.063	4.157	0.046			
Gender	[-0.323, 0.185]						-0.069	0.127	0.590
Age	[0.005, 0.513]						0.259^∗^	0.127	0.046
Model 3		0.435	0.190	0.108^∗∗^	8.025^∗∗^	0.006			
Gender	[-0.332, 0.151]						-0.091	0.121	0.455
Age	[0.012, 0.493]						0.253^∗^	0.110	0.040
Autonomy Priming	[0.097, 0.563]						0.330^∗∗^	0.116	0.006


**N* = 64. ^∗^*p* < 0.05; ^∗∗^*p* < 0.01.*

## General Discussion

By using a modified illusory choice paradigm (adapted from Huang et al., 2009) to measure the mere choice effect the current research examined how autonomy would affect the choice effect even when the actual choice did not occur. Replicating the previous findings (Huang et al., 2009), the perceived choice, without involving a real choosing process, has also been found to enhance the attractiveness of an object in a autonomy-sufficient condition (Studies 1 and 2), which is termed as the choice effect. The sense of autonomy was measured not only as a trait by using questionnaire (Study 1), but also as a state by setting a priming task of recall writing (Study 2).

Our hypothesis that autonomy increases the choice effect was supported both when autonomy was measured as an individual-difference variable (Study 1) and when it was experimentally manipulated (Study 2). In Study 1, the level of trait autonomy was positively related with the choice effect. In Study 2, when state autonomy was enhanced, participants displayed a larger choice effect. When primed by the autonomy fulfillment recalling task, participants rated their chosen objects as more favorably than the objects chosen by others. That is to say, the choice effect occurred after one’s state autonomy had been induced (see Study 2, in the autonomy fulfillment condition). Consistent with previous findings (Huang et al., 2009), we also found that the choice effect appeared without any autonomy related treatment (see Study 2, in the control condition). Interestingly, the choice effect disappeared when participants were primed with state autonomy deprivation (see Study 2, in the autonomy deprivation condition). The two studies suggested that autonomy fulfillment is the premise of the choice effect, such that if people experience autonomy deprivation, their choice-induced preference would decrease or would even disappear.

Choice-induced preference has been a topic of longstanding interest in social psychology (Brehm, 1956; Steele, 1988; Lieberman et al., 2001; Gawronski et al., 2007; Huang et al., 2009; Egan et al., 2010). In the objects evaluation IAT task of the current study, the choice effect holds that people have a more positive attitude toward an object merely because they perceive choice of it. The perceived choice itself is sufficient to induce such effect. This evidence supports that choices influence preferences through a natural and automatic process, and the choice-induced preference is a byproduct of the choice (Leotti et al., 2010).

The occurrence of the mere choice effect is possibly related to the many aspects of the self-concept, such as self-serving or self-protecting biases (e.g., Sedikides and Strube, 1997), self-enhancement (e.g., Kurman, 2001), self-affirmation (e.g., Brown and Dutton, 1995), and self-verification (e.g., Chen et al., 2006). According to self-enhancement theory, people over evaluate self-related issues to maintain a positive self-image (e.g., Kurman, 2001). As “my choice” is a part of the self-concept, the positive words that describe the self-chosen objects represent the positive valence of self node (Greenwald et al., 2002). People experience the more positively self-image in choice effect, due to that “my choice” is given positive postchoice ratings. Faced with the need to maintain a positive self-image, participants would evaluate the “self-chosen” objects over the “non-self-chosen” objects, and that would result in the choice effect.

The most intriguing and main finding in the current study is that this choice effect was affected by the sense of autonomy. As showed in the results, a lager choice effect was elicited in the participants that experienced state autonomy fulfillment rather than in those with no priming treatment, but the trend did not reach a significant level. In addition, the choice effect disappeared when participants experienced autonomy deprivation. In the perceived choice-preference link, people’s favorability on the self-chosen objects in the state autonomy fulfillment condition remains as high as in the control condition, whereas this favorability would be weakened and would even disappeared if they experienced autonomy deprivation. The evidence that trait autonomy is positively correlated with the choice effect is consistent with this finding. In a word, autonomy moderated the relationship between the perceived choice and the induced preference.

The mere exercise of choice itself is assumed to provide a sense of autonomy (e.g., Iyengar and Lepper, 1999). People evaluate the chosen alternative as more desirable than the rejected alternative, in order to reassert their autonomy (Hammock and Brehm, 1966). The sense of autonomy, which has been treated as the expression or a result of actual choosing behavior, fulfills important psychological functions, such as enhancing happiness (Chekola, 2007; Demir et al., 2011) and increasing subjective well-being (Sheldon et al., 2004). Thus, people perceiving choice may experience the sense of autonomy, which will generate a positive feeling on the self-chosen objects, and that in turn will enhance the evaluation of the objects. Experiencing autonomy, which makes people feel free to act their own decisions, would improve individuals’ feeling of the self-image. Besides, compared to those in who were merely aware of the choice, individuals who were primed with autonomy fulfillment displayed only a relatively, but not significantly larger trend in choice induced preference, because merely perceiving the choice could elicit the autonomy experience. The beforehand autonomy priming only contributes a little bit more on the basis of the autonomy experience induced by the choice.

One thing that needs to be pointed out in our objects evaluation IAT task is that participants were given the choosership and assigned to specific objects. That is, the perceived choice assigned to participants was not actually based on their free will. The autonomy induced by mere choice may be weaker than that induced by actual choice. Assuming that one’s trait autonomy is stable, although the subsequent object evaluation task may elicit autonomy, this level could be canceled out by the previously primed autonomy deprivation. When the sense of autonomy has been deprived, one’s intrinsic motivation and sense of control decreases (Zuckerman et al., 1978; Simon and McCarthy, 1982, Unpublished), and that generate a negative feeling on self-chosen objects, which in turn impairs the object-evaluation.

Taken together, this study found new evidence to explain the mechanism of the choice effect. That is, the sense of autonomy affects the choice effect, in other words, experiencing autonomy moderated the choice-preference link.

Although our study tapped on the mechanism underlying choice-induced preferences, the results still bear on some limitations. First, we did not directly test the positive and negative attitude on the self-chosen and the other-chosen objects separately. We just combined the attitudes to the positive-self-chosen objects with that to the negative-other-chosen ones, and the attitudes to the negative-self-chosen objects with that to the positive-other-chosen ones. In the future study, we could separate them and measure the attitude to one’s positively or negatively described objects separately by recording the real-time brain activities, which could also provide an implicit way measuring the attitude. Second, we did not record participants’ explicit preference on objects, but only used the implicit attitudes as our indicator of preference. Although attitude on objects was evaluated in an implicit way by an IAT, which has the advantage of being immune to demanding characteristics and social desirability, it is necessary to replicate our findings using other explicit paradigm to confirm that the result can be generated in different kinds of situations. Third, we used the scenario in which participants were told which objects they have chosen, but not the actual choice action. A previous related study (Huang et al., 2009) using the same paradigm provided the evidence on the existence of a mere choice effect. Although this previous study has already verified that the virtual choice has the same efficacy as the actual choice. To be more carefully considered, we have to admit that the possible explanation is the vignette format. To fully verify the robust relationship of autonomy and choice effect, future research should investigate whether or not the actual choice actions provide a stronger relationship than that in the assigned choice. The relationship of autonomy deprivation and choice effect would be strengthened in the actual choice actions rather than assigned choice settings because of the more efforts in actions.

The findings of the current research reveal that autonomy affects the mere choice effect: (1) individual’s autonomy trait is positively correlated with the mere choice effect; (2) the experience of autonomy deprivation decreases the mere choice effect, which results in that people do not valuate self-chosen objects more favorably than other-chosen objects anymore. Our research provides good insights in the relationship between autonomy and the mere choice effect, and contributes to the theoretical understanding of the mechanism in choice-induced preferences.

## Author Contributions

LW proposed the main research idea. TT, ZS, and LW made the research design. TT ran the experiments. TT and ZS did the statistic analysis. ZS and LW wrote the manuscript.

## Conflict of Interest Statement

The authors declare that the research was conducted in the absence of any commercial or financial relationships that could be construed as a potential conflict of interest.
